# Beach Buffet: First Observations of White‐Backed Vultures *Gyps africanus* Feeding on a Cape Fur Seal *Arctocephalus pusillus* on the Skeleton Coast

**DOI:** 10.1002/ece3.73213

**Published:** 2026-03-08

**Authors:** Ruben Portas, Ortwin Aschenborn, Piet Beytell, Mark Boorman, Holger Kolberg, Joerg Melzheimer, Emsie Verwey, Miha Krofel

**Affiliations:** ^1^ Biotechnical Faculty University of Ljubljana Ljubljana Slovenia; ^2^ Leibniz Institute for Zoo and Wildlife Research Department of Evolutionary Ecology Berlin Germany; ^3^ School of Veterinary Medicine University of Namibia Windhoek Namibia; ^4^ Ministry of Environment, Forestry and Tourism Directorate of Scientific Services Windhoek Namibia; ^5^ Vultures Namibia Swakopmund Namibia; ^6^ Hoanib Skeleton Coast Camp Research Centre, Wilderness Windhoek Namibia

**Keywords:** carcass, *Gyps africanus*, marine carrion, scavenging, vultures

## Abstract

Vultures are avian obligate scavengers which provide important ecosystem services by efficiently removing carcasses from the landscape. Some species are now being observed feeding in coastal environments where they consume marine carcasses (i.e., whales, dolphins, seals and fish). On the African continent, only two species thus far have been reported to feed in the coastal ecosystems: the Lappet‐faced Vulture (*Torgos tracheliotos*) on the Skeleton Coast of Namibia and the Hooded Vulture ( *Necrosyrtes monachus* ) in southern Gambia. Here we report on the first observation of four White‐backed Vultures feeding on a Cape fur seal ( *Arctocephalus pusillus* ) killed by lions (*Panthera leo*). These observations carry several ecological and conservation implications and raise certain conservation concerns, including marine nutrients transfer and potential accumulation of marine environmental toxicants.

## Graphical abstract

Vultures are specialized scavengers that provide critical ecosystem services by clearing carcasses, though their activity in African coastal environments has previously been limited to only two documented species (i.e., the Lappet‐faced and the Hooded Vultures). This report documents the observation of White‐backed Vultures feeding on Cape fur seals, marking a significant expansion in the known foraging behavior of this species. These findings highlight important conservation considerations, specifically regarding the transfer of marine nutrients to terrestrial ecosystems and the potential risks associated with the exposure of vultures to the accumulation of marine‐derived toxins.

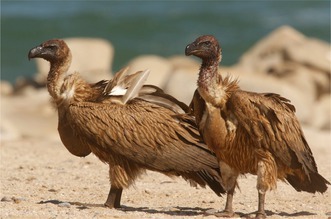

## 1 Introduction

African vultures are avian obligate scavengers which feed predominantly on ungulate carcasses and other large vertebrate mammals (Mundy 1982). They soar over wide ranges searching for carcasses (i.e., 50 to 200 km per day at cruising altitudes of 250 to 500 m) (Mundy et al. 1992; Phipps et al. 2013; Spiegel et al. 2013), on which they feed, and their consumption of carcasses is considered a crucial ecosystem service that aids in preventing disease outbreaks (Buechley and Sekercioglu 2016). So far, only two of the nine African vulture species have been reported to feed on marine carrion: the Hooded Vulture ( *Necrosyrtes monachus* ) in the south of Gambia (Barlow et al. 2021) and the Lappet‐faced Vulture (*Torgos tracheliotos*) on the Skeleton Coast of Namibia (P. Bridgeford 2023). While observations of Lappet‐faced Vultures are restricted to feeding on Cape fur seals ( *Arctocephalus pusillus* ) (P. Bridgeford 2023), Hooded Vultures were recorded feeding on a variety of marine species, including pinnipeds, cetaceans, fish, crabs and marine birds (Barlow et al. 2021). In the Americas, the Andean Condor ( *Vultur gryphus* ) (Lambertucci et al. 2018), the California Condor ( *Gymnogyps californianus* ) (Chamberlain et al. 2005; Herring et al. 2018) and the Black and Turkey Vultures ( *Cathartes aura* and *Coragyps atratus* ) (Pavés et al. 2008; Blazquez et al. 2016; Gunderson 2018) have been recorded feeding on marine carrion. Since levels of environmental toxicants increase exponentially with each trophic level (Krüger et al. 2022; Ottinger and Dean 2022), this could represent a threat for vulture health. Despite its relevance for the trophic cascades and the exposure of these threatened species to environmental contaminants present in the marine ecosystems (Mee et al. 2007; Herring et al. 2018; Krüger et al. 2022; Erasmus et al. 2024), vultures feeding on marine carrion have so far received little attention in the African continent.

The White‐backed Vulture ( *Gyps africanus* ) is listed as a Critically Endangered species (Ogada et al. 2015; BirdLife International 2021) and despite being the most widespread African vulture species, its population has declined around 80% within the last four decades (BirdLife International 2021). White‐backed Vultures are gregarious birds that favor savannah landscapes. Here, we report on a unique observation of four White‐backed Vultures feeding on a Cape fur seal (*Arctocehalus pusillus*) carcass in the Skeleton Coast of Namibia. In addition, we discuss the ecological and conservation relevance of the observation which opens opportunities for future research.

## 2 Methods

The observation took place at the Möwe Bay seal colony (19.37° S, 12.71° E), in the Skeleton Coast National Park (SCNP), northwest of Namibia while the first author was visiting clusters of GPS positions of Lappet‐faced Vultures fitted with satellite GPS trackers. When vultures of different sizes and color tones were detected, the group was approached carefully by vehicle with frequent stops to not disturb them and gather further observational data from the car. The whole observation was conducted using Swarovski 10 × 40 binoculars and pictures taken with a Canon DSLR equipped with a 100–400 mm lens. Individuals were assigned to the immature age class due to the lack of white in the lower back and rump diagnostic of adult individuals and the presence of darker brown streaked plumage typical of immature White‐backed Vultures. There is little vegetation around the Möwe Bay seal colony, and it is mostly dominated by the characteristic succulent vegetation of the coastal Namib Desert (Giess 1968). The average annual precipitation is less than 50 mm and it is mostly in the form of coastal fog, which occurs predominantly during the cold‐dry (approximately May to August) and hot‐dry (approximately September to January) seasons (Seely et al. 1998). The minimum average monthly temperature ranges from 8°C to 10°C and the maximum average temperature ranges from 26°C to 28°C (Atlas of Namibia Team 2022). In the days prior to the observation, the east wind had been blowing toward the coast at speeds up to 50 km/h (https://weatherspark.com/h/m/76231/2025/6/Historical‐Weather‐in‐June‐2025‐in‐Walvis‐Bay‐Namibia#Figures‐WindSpeed) and with hot temperatures (averaging 30°C).

## 3 Results

**FIGURE 1:**
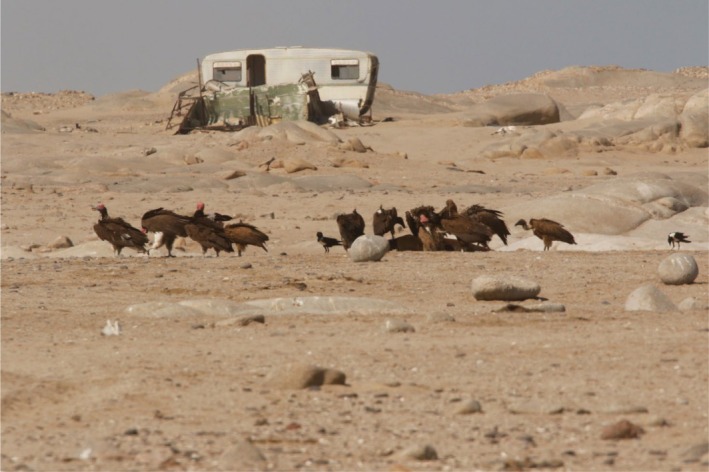
A group of eight Lappet‐faced Vultures and three White‐backed Vultures gathered around a seal carcass on the Atlantic coast in the Skeleton Coast National Park, Namibia. Another group of vultures was located outside of the picture frame to the left. At the back is a caravan once used by diamond prospectors that has now become part of the national park landscape. Photo: R. Portas.

**FIGURE 2:**
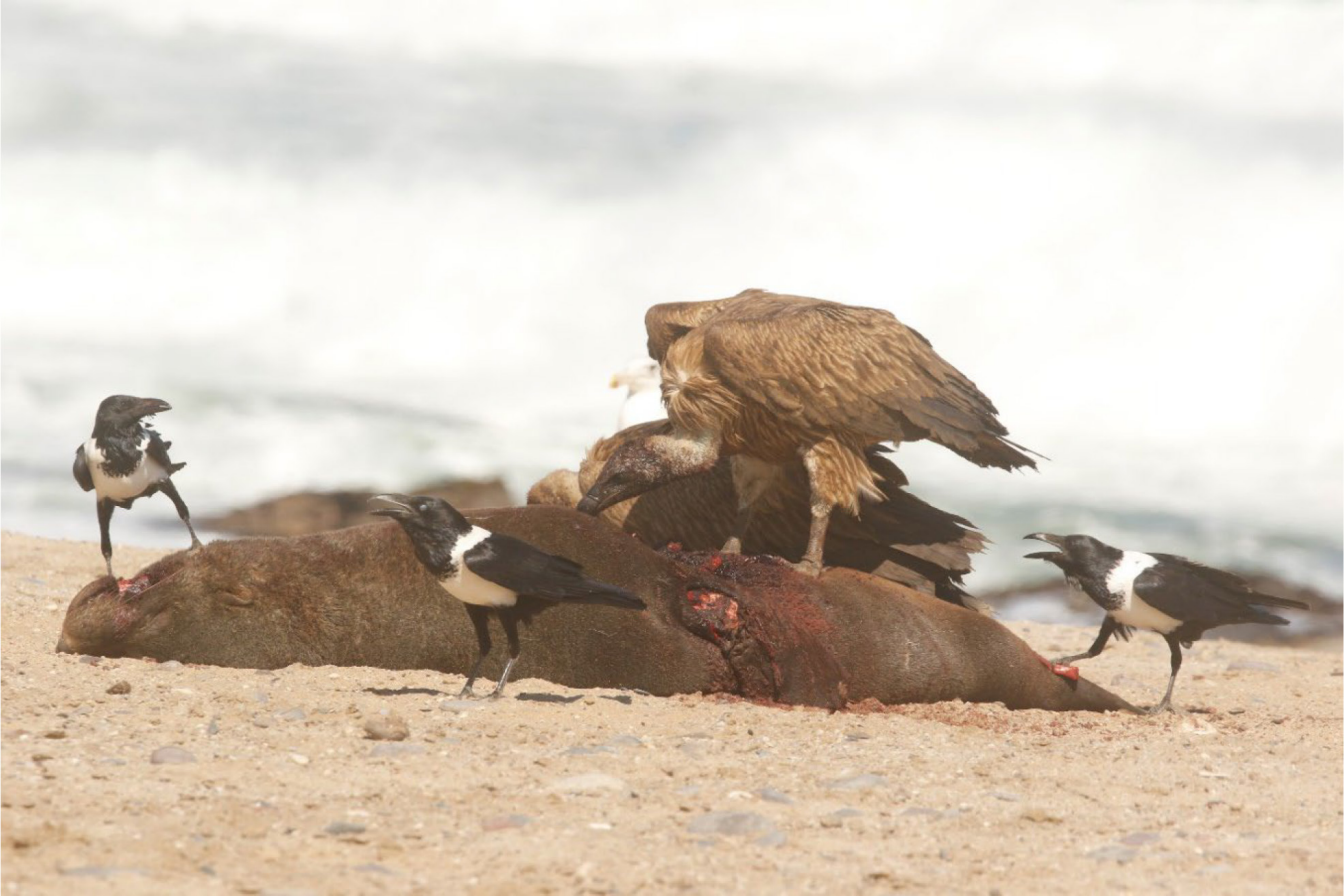
Two White‐backed Vultures remained feeding on the seal carcass surrounded by Pied Crows with a Kelp Gull in the background. Photo: R. Portas.

**FIGURE 3:**
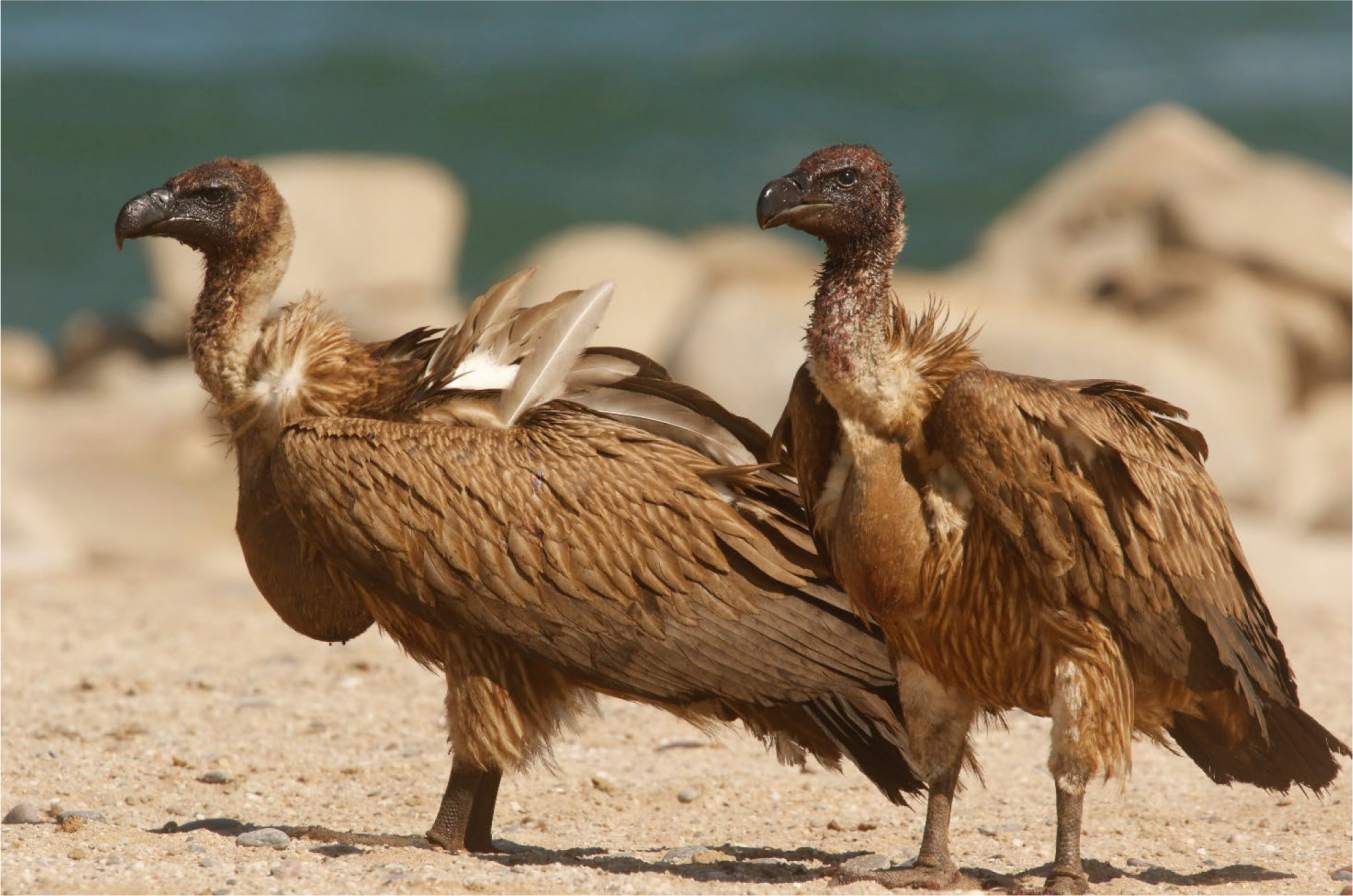
Two White‐backed Vultures show their full crops of stored food after feasting on the seal carcass. Photo: R. Portas.

On the 13th of June 2025, a group of 17 vultures was sighted feeding on an adult female Cape fur seal carcass freshly killed by lions (*Panthera leo*) the previous night on the beach in Möwe Bay in the SCNP (Figure 1). The group was initially composed of 13 Lappet‐faced Vultures, of which 11 were adults and 2 immatures. The other four vultures were White‐backed Vultures, all of them immature birds. The seal carcass was located a few meters away from the track driven by tour guides visiting the seal colony earlier and approximately 100 m from the shore. Lion footprints and drag marks from the carcass were seen at the scene. The observation lasted approximately 1 h, during which some of the Lappet‐faced Vultures walked away and flew to neighboring rocky outcrops, and two White‐backed Vultures remained feeding at the carcass surrounded by Pied Crows ( *Corvus albus* ) and Kelp Gulls ( *Larus dominicanus* ) (Figure 2 and Video S1). After collecting photographic and video footage, the car was driven away, and six of the Lappet‐faced Vultures returned to the carcass. In the footage, several birds can be seen with full crops (Figure 3).

## 4 Discussion

We report here, to our knowledge, the first observation of White‐backed Vultures feeding on marine carrion. The species has been increasing its presence in the Namib Desert over the last 10 years and during the 2024 vulture breeding survey carried out, four nests were found inside the Namib Naukluft National Park (Vultures Namibia unpublished data) which is 170 km from the nearest seal colony where Vulture presence has been recorded. A single adult bird was seen twice feeding on a Springbok ( *Antidorcas marsupialis* ) carcass approximately 50 km inland from Möwe Bay. Therefore, it is possible that White‐backed Vultures do sporadically travel along the Namibian coast but their activity has not yet been not detected due to the low human population density and the remoteness of this seal colony.

In contrast, Lappet‐faced Vultures were first reported feeding at the seal colony in 2004 (P. Bridgeford 2023) and have since become a regular sight at the seal colonies in the north of the Skeleton Coast (i.e., Angra Fria, Möwe Bay and Toscanini). However, we are not aware of any sightings of these birds at other seal colonies of central Namibia (i.e., Cape Cross and Walvis Bay) which are regularly frequented by human observers or in the southern coast of the country (i.e., Wolf Bay, Atlas Bay, Van Reenen Bay and Baker's Bay) (Marie Lemerle, pers. comm.). As such, vultures feeding on marine food sources and flying along the coast seems currently confined to the SCNP. In the days leading up to our observation, steady easterly winds of roughly 50 km/h were blowing from the inland toward the coastline. Easterly winds in Namibia have been associated with increased sightings of vagrant birds in coastal towns (see *Lanioturdus*: https://www.namibiabirdclub.org/#archive). Wind can have a strong effect on bird flight as previously described in studies of soaring flight and wind support (Safi et al. 2013; Brooks 2024). We hypothesize that the four vultures we observed may have been carried beyond the dune belt onto the barren coastal landscape by these winds. Since adult individuals generally outperform juveniles under challenging flight conditions, particularly when wind shear and thermal variability are present (Harel et al. 2016), the fact that all of the individuals we observed were immatures could indicate a wind‐related impact in their flight trajectories. Vultures in their immature life stages exhibit high spatial plasticity and often explore new areas in search of unpredictable trophic resources (Mundy et al. 1992; Phipps et al. 2013). It is therefore plausible that the four observed individuals found the seal carcass during an exploratory foraging trip. The observation we describe is currently an isolated report. However, as previously mentioned, after their initial sighting in 2004, Lappet‐faced Vultures were observed at the coast for the first time and have since become a regular sight at the seal colonies of the SCNP. This may also occur with the White‐backed Vulture population following the discovery of this new food source by the four individuals reported here. Cape fur seals are a common prey item from a limited number of lions living along the northern Namibian coast (P. A. Bridgeford 1985; Stander 2019). The presence of a broader scavenger and carnivore guild (e.g., lions, Black‐backed jackals (*Lupullela mesomelas*) and Lappet‐faced vultures) may as well facilitate White‐backed vultures to access to the seal flesh by opening the carcasses and therefore becoming an optimal food source. The permanent establishment of lion prides along the SCNP feeding on marine items and predating on adult seals (Stander 2019) now provides a regular supply of fresh carcasses on the beach. This food source is thus no longer limited to the austral summer seal breeding season, which only provides a pulsed resource of seal pup carcasses restricted in space and time (to seal colonies and seal breeding season, respectively). These dynamics have important ecological implications and open the door to future research, particularly studies on the transfer of marine‐derived nutrients by scavengers into the desert ecosystems.

This marine–terrestrial linkage also potentially exposes vultures to a range of environmental contaminants that bioaccumulate within the prey tissues and biomagnify across trophic levels (Kurle et al. 2016; Krüger et al. 2022). Of particular concern are heavy metals—such as mercury (Hg), lead (Pb) and cadmium (Cd)—already detected in California condors (Wiemeyer et al. 1988; Kurle et al. 2016), alongside persistent organic pollutants (POPs) previously found in Californian condors (Kurle et al. 2016) and pinnipeds (Blasius and Goodmanlowe 2008) and halogenated organic compounds (HOC) (Stack et al. 2022). Furthermore, the detection of Type A influenza viruses in marine birds along the Namibian coastline poses a significant biosecurity risk to the avian scavenger guild (Molini et al. 2020, 2023). The ingestion of these toxical and pathogenic agents could hinder the reproductive success and weaken immune system of these threatened avian scavengers (Herring et al. 2018; Krüger et al. 2022). Given the precarious conservation status of all vulture species, further research is urgently required.

## Author Contributions

**Ruben Portas:** conceptualization (lead), data curation (lead), funding acquisition (equal), investigation (lead), methodology (equal), project administration (equal), writing – original draft (lead), writing – review and editing (equal). **Ortwin Aschenborn:** funding acquisition (supporting), project administration (supporting), validation (equal), writing – review and editing (equal). **Piet Beytell:** funding acquisition (supporting), validation (equal), writing – review and editing (equal). **Mark Boorman:** funding acquisition (supporting), validation (equal), writing – review and editing (equal). **Holger Kolberg:** funding acquisition (supporting), validation (equal), writing – review and editing (equal). **Joerg Melzheimer:** funding acquisition (supporting), project administration (supporting), resources (supporting), validation (equal), writing – review and editing (equal). **Emsie Verwey:** validation (equal), writing – review and editing (equal). **Miha Krofel:** funding acquisition (lead), project administration (lead), supervision (supporting), writing – review and editing (equal).

## Funding

This study was supported by the Slovenian Research and Innovation Agency (grant J1‐50013).

## Conflicts of Interest

The authors declare no conflicts of interest.

## Supporting information


**Data S1:** Picture Cover White Backed Vulture_Ruben Portas.

**Data S2:** White backed vulture feeding on cape fur seal.

## Data Availability

The full scope of the data is presented within the figures and video materials and is available to the public.
