# Supplementary figures and images for: Beach Buffet: First Observations of White‐Backed Vultures Gyps africanus Feeding on a Cape Fur Seal Arctocephalus pusillus on the Skeleton Coast
Source: Ecol Evol. 2026 Mar 8;16(3):e73213. doi: 10.1002/ece3.73213 (PMC12968060; doi:10.1002/ece3.73213)

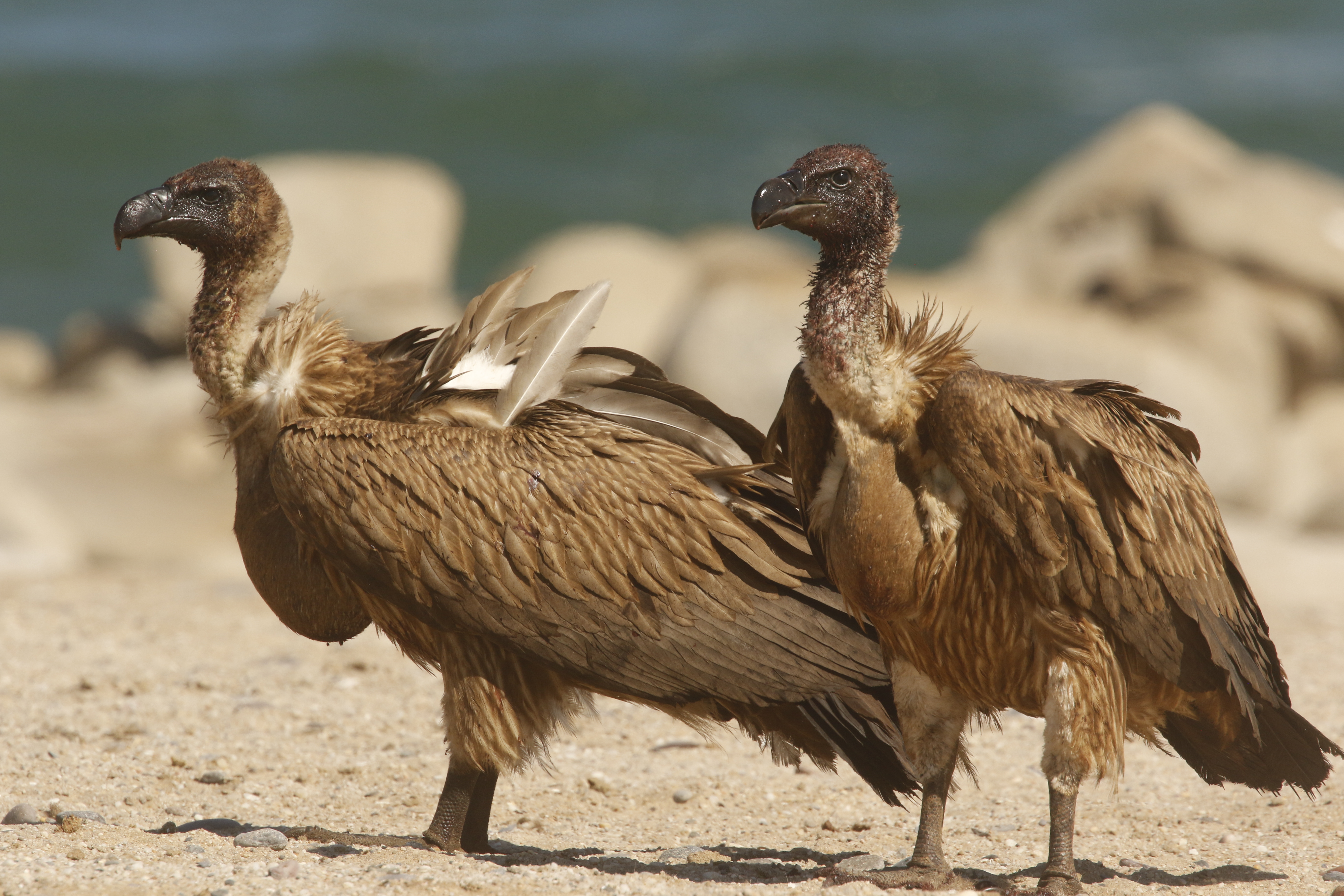

Supplement: Supplementary file 1 — Data S1: Picture Cover White Backed Vulture_Ruben Portas. [file ECE3-16-e73213-s002.jpg]
